# Preoperative platelet lymphocyte ratio as independent predictors of prognosis in pancreatic cancer: A systematic review and meta-analysis

**DOI:** 10.1371/journal.pone.0178762

**Published:** 2017-06-02

**Authors:** Wei Song, Chuan Tian, Kai Wang, Run-jin Zhang, Shu-bing Zou

**Affiliations:** 1 Department of Hepatobiliary Surgery, The Second Affiliated Hospital of Nanchang University, Nanchang, China; 2 Department of Nuclear Medicine, Guizhou Provincial People’s Hospital, Guiyang, China; Virginia Mason Medical Center, UNITED STATES

## Abstract

**Background:**

Recently, the preoperative platelet to lymphocyte ratio (PLR) has been found reported to predict oncologic outcomes in multiple malignancies. However, its prognostic value in patients with pancreatic cancer (PC) remains controversial. The aim of this study was to assess the prognostic value of preoperative PLR in PC.

**Methods:**

MEDLINE, EMBASE, and Cochrane databases were searched to identify studies evaluating the prognostic significance of preoperative PLR in PC. Pooled hazard ratios (HRs) for overall survival (OS) were calculated using fixed-effects/random-effects models.

**Results:**

A total of eight studies comprising 1,904 patients with PC were included in the meta-analysis. The pooled analysis demonstrated that elevated PLR had an association with decreased OS (HR: 1.22, 95% CI: 1.04–1.43, p = 0.02). Subgroup analysis showed that a high PLR significantly predicted poor OS in Asian studies (HR: 1.25, 95% CI: 1.03–1.52, p = 0.02), patients with metastatic disease (HR: 1.34, 95% CI: 1.01–1.77, p = 0.04) and patients with PLR >150 (HR: 1.73, 95% CI: 1.21–2.49, p = 0.003).

**Conclusions:**

The preoperative PLR may be a significant independent prognostic factor in patients with PC.

## Introduction

Pancreatic cancer (PC) is the fourth leading cause of cancer-related death worldwide [1]. Radical resection without residual tumor is the most effective therapy for the majority of patients. Nevertheless, more than 80% of patients are diagnosed at inoperable late stages [2], and the prognosis is extremely poor. Data from the Surveillance, epidemiology and End Results (SEER) database (2006–2012) demonstrates that the 5-year survival of patients with pancreatic cancer is 7.7%. Therefore, identifying a predictive biomarker that could be used to determine individualize therapy and to predict prognosis remains important.

Host inflammatory responses can largely influence tumor development and progression [3]. Several inflammatory factors, such as plasma fibrinogen, neutrophil to lymphocyte ratio (NLR), lymphocyte to monocyte ratio (LMR) are identified as useful indicators for predicting the prognosis in renal cell carcinoma, ovarian cancer, and hepatocellular carcinoma [4–6]. Recently, the preoperative platelet to lymphocyte ratio (PLR), which also reflects the degree of systemic inflammation, has been found to be linked to prognosis in patients with PC [7–9]. However, some studies failed to find correlation between PLR and prognosis of PC [10–12]. We therefore conducted a meta-analysis to assess the prognostic effect of preoperative PLR in PC.

## Materials and methods

### Search strategies

The present study was performed in accordance with the preferred reporting items for systematic reviews and meta-analyses guidelines [13]. We searched MEDLINE, EMBASE, Cochrane databases from inception up to August 2016. The following search terms were used: “pancreatic cancer” or “pancreatic ductal adenocarcinoma”, “platelet to lymphocyte ratio” or “PLR” or “platelet lymphocyte ratio” or “platelet-lymphocyte ratio”, “prognostic” or “prognosis” or “survival” or “outcome”. In addition, manual searches were performed in the web and by reviewing the citation lists of the retrieved articles. However, we did not search the grey literature. Detailed search strategies refer to S2 File.

### Study selection

The criteria for inclusion were listed as follows: (1) PC was pathologically confirmed; (2) assessing the prognostic value of preoperative PLR on OS; (3) studies supplied sufficient information for calculating hazard ratio (HR) and 95% confidence interval (CI); and (4) reporting a dichotomous cut-off value for PLR. The exclusion criteria were as follows: (1) reviews, letters, case-reports, and conference abstracts without original data; (2) overlapping or duplicate data; and (3) non-English language studies.

### Data extraction

The two reviewers independently reviewed all eligible studies and extracted data. Any disagreement was resolved by discussions among all coauthors. The following information was collected: first author’s name, year of publication, country, number of patients, region of publication, tumor stage, overall survival (OS), survival analysis methods, cut-off values, and time of follow-up. HRs were extracted from multivariate or univariate analyses or estimated from Kaplan-Meier survival curves [14].

### Quality assessment

The quality of each study was assessed in accordance with the Newcastle-Ottawa Scale (NOS) [15], which included an assessment of subject selection, comparability of groups, and clinical outcome. A total of nine items were extracted, and each item scored 1. The total scores ranged from 0 to 9. If scores are ≥7, the study is considered as high quality.

### Statistical analyses

The meta-analysis was conducted by STATA 12.0 (College Station, TX, USA). Heterogeneity of the HR of each study was quantified using Cochran’s Q test and Higgins-I^2^ statistic. A p-value < 0.1 for the Q-test or I^2^ >50% was considered statistically significant. A random effect model was used if heterogeneity was observed, while a fixed effect model was applied in the absence of inter-study heterogeneity. HRs and corresponding 95% CIs were directly extracted from the published data or calculated using previously published methods proposed by Tierney et al. [16]. Subgroup analyses were conducted based on the region of publication, tumor stage, treatment method, NOS score, and the cut-off value of PLR. Sensitivity analyses were carried out to evaluate result stability excluding each study. If the number of included studies was more than 11, the publication bias was evaluated using funnel plots and with the Begg’s funnel plots and Egger’s tests [17, 18]. P < 0.05 was considered statistically significant.

## Results

### Search results

**Fig 1:**
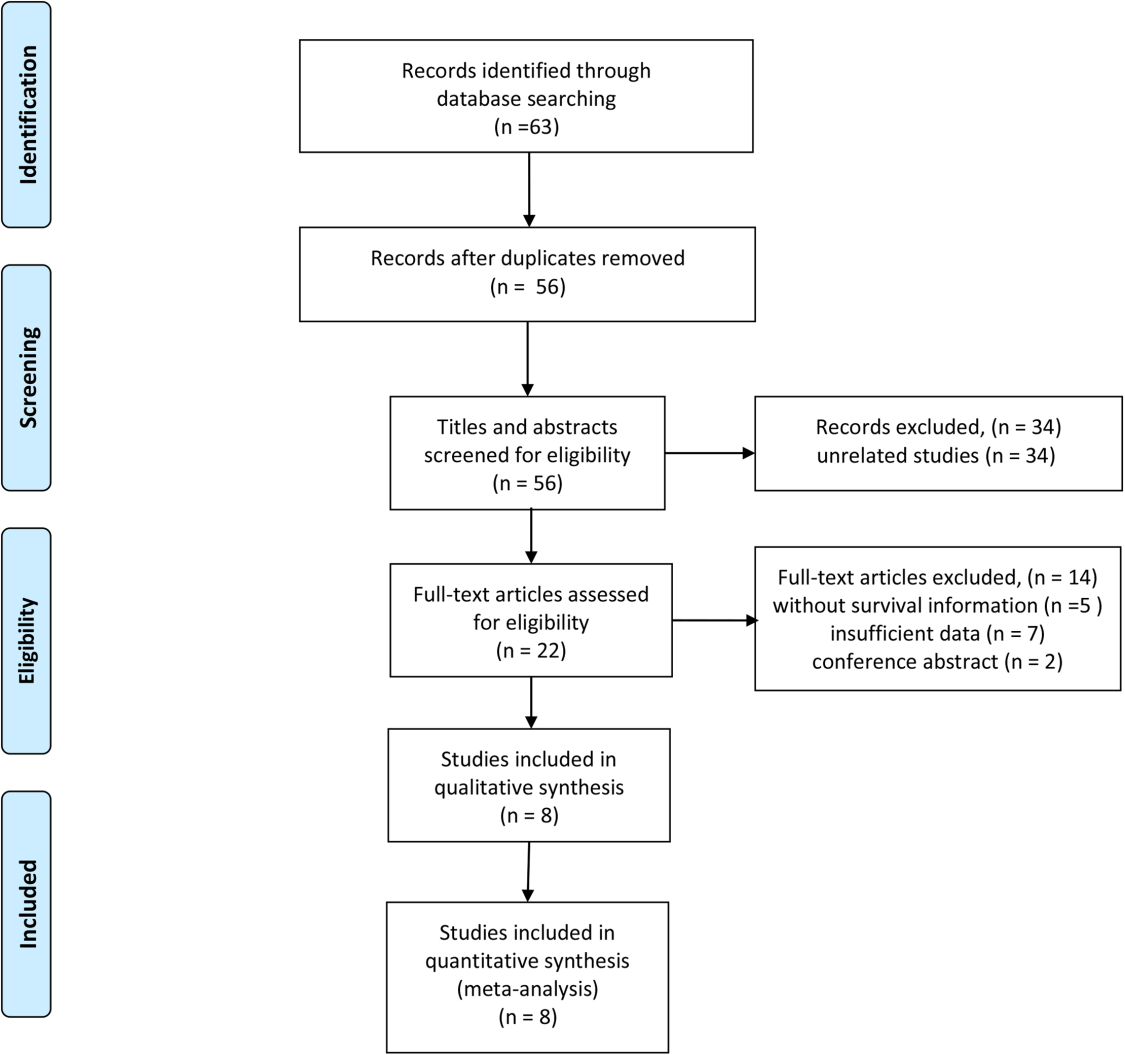
Flow diagram of the study selection process.

Our search strategy yielded 63 potentially relevant articles. After removing duplicates, 56 articles remained to be screened. Of these, 34 were excluded through titles and abstracts, leaving 22 articles for detailed evaluation. As a result, 8 eligible studies, comprising a total of 1,904 patients, were included in the quantitative synthesis. The selection process was shown in Fig 1.

### Characteristics of the included studies

Most of these studies have been published since 2014. The sample sizes ranged from 110 to 440. Six studies reported on Asians, and two study on Europeans. HRs and 95% CI were extracted directly from the seven studies. HRs in one study were estimated by Kaplan-Meier survival curves. The cut-off values for PLR ranged from 126 to 225, two studies used a PLR cut-off value ≥ 150, while six studies used a PLR < 150. In methodological quality of studies, the overall NOS scores ranged 6 to 8, with a median of 6.8. Table 1 lists the detailed study characteristics.

**Table 1:** Characteristics of the studies included in the meta-analysis.

Author	Year	Country	Region	Follow-up (months)	Treatment	No. of patients	Stage	Cut-off value	Survival analysis	Analysis	NOS score
Shirai	2015	Japan	Asia	NA	Surgery	150	No-metastatic	150	OS	MV/UV	6
Smith	2009	UK	Europe	12(7.8–25.5)	Surgery	110	No-metastatic	150	OS	MV	6
Qi	2015	China	Asia	NA	Chemotherapy	211	Metastatic	126	OS	MV/UV	7
Martin	2014	Austria	Europe	12	Mixed	124	Metastatic	200	OS	MV/UV	8
Kou	2016	Japan	Asia	10.8(1.7–72.1)	Chemotherapy	306	Metastatic	150	OS	UV	7
Lnoue	2014	Japan	Asia	18.7(6.1–68.2)	Mixed	440	Mixed	150	OS	MV/UV	8
Asari	2016	Japan	Asia	18(10–35)	Surgery	184	Mixed	225	OS	MV/UV	6
Yamada	2016	Japan	Asia	15.1(0.43–150.7)	Surgery	379	Mixed	150	OS	MV/UV	8

OS: overall survival; MV: multivariate; UV: univariate; NA: not available

### Meta-analysis

#### Overall survival

**Fig 2:**
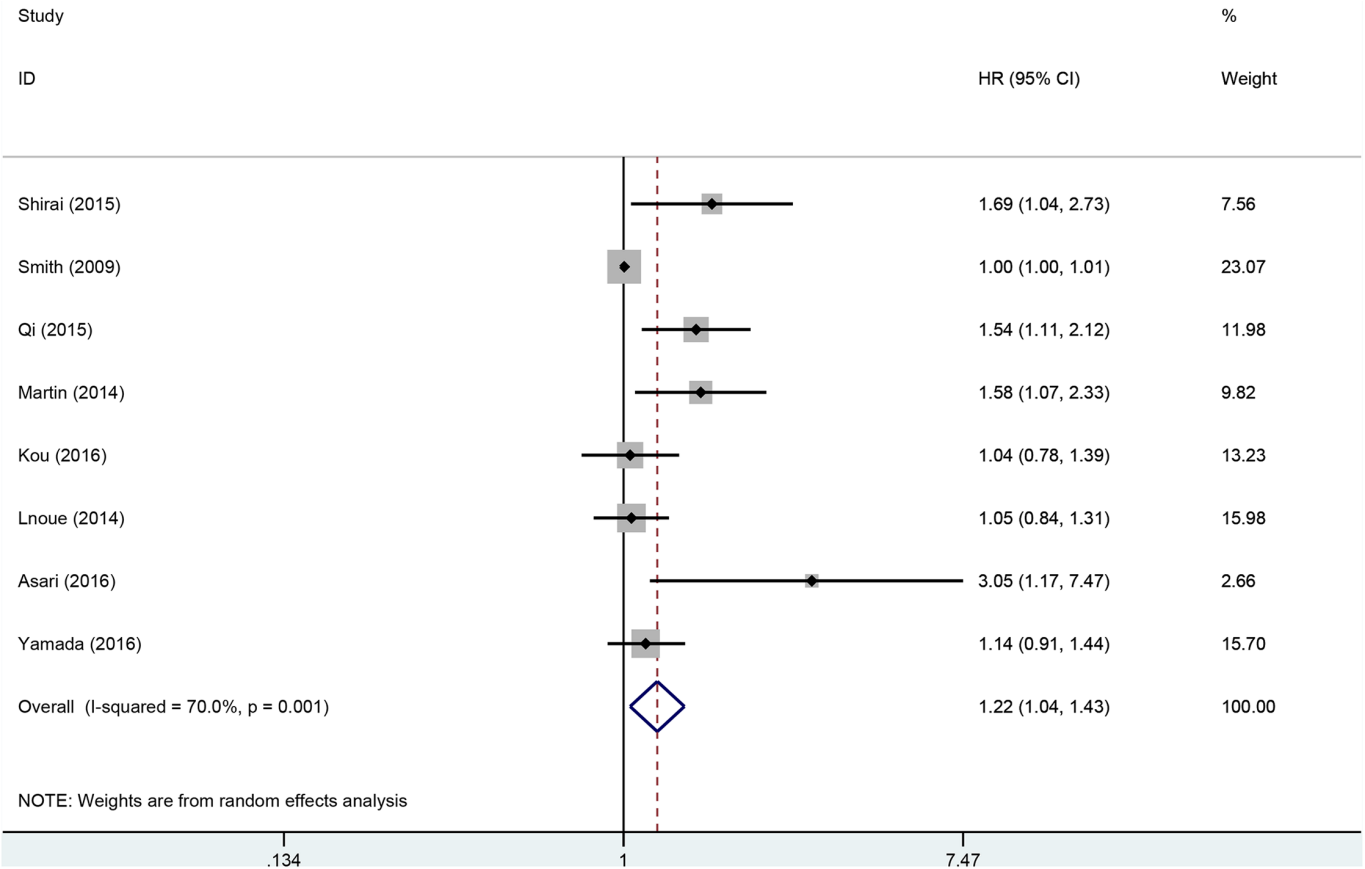
Forest plots for the association between PLR and OS.

A total of 1,904 patients from eight studies were included in the analysis of HRs for OS. The pooled analysis demonstrated that elevated PLR had an association with decreased OS (HR: 1.22, 95% CI: 1.04–1.43, p = 0.02). However, excessive heterogeneity existed between studies (p = 0.001, I^2^ = 70%). Thus, the random-effects model was used (Fig 2).

To detect the potential heterogeneity, subgroup analyses stratified by region of publication, treatment, disease stage, NOS score, and the cut-off value of PLR (Table 2). Exploratory subgroup analysis according to region of publication showed that high PLR predicted worse OS in Asian studies (HR: 1.25, 95% CI: 1.03–1.52, p = 0.02). When stratified by treatment methods, high PLR did not have prognostic effect in all subgroups. Pooled HRs for OS stratified by disease stage, elevated PLR predicted decreased OS in patients with metastatic disease (HR: 1.34, 95% CI: 1.01–1.77, p = 0.04). The cut-off values ranged from 126 to 225. We stratified cut-off values into two subgroups: ≤150 and >150. Stratification by the cut-off value found that patients with PLR >150 had significantly worse OS (HR: 1.73, 95% CI: 1.21–2.49, p = 0.003), however, the prognostic effect disappeared in patients with PLR ≤150 (HR: 1.13, 95% CI: 0.99–1.30, p = 0.08). Subgroup analysis according to NOS score found that the prognostic role of PLR was observed in studies with NOS score ≥7 (HR = 1.18; 95% CI = 1.04–1.33; P = 0.008), however, when NOS score <7, the prognostic efficiency disappeared in the pooled results. In order to assess the influence of single studies on the overall estimate, the sensitivity analysis was performed. Each single study was removed each time to estimate the influence of individual data sets on the combined HR for OS. The results showed that no study had a significant effect on the observed effect size (pooled HR), indicating the robustness of our findings.

**Table 2:** Pooled hazard ratios (HRs) for OS according to subgroup analyses.

Subgroup	No. of studies	No. of patients	Effects model	HR (95% CI)	P value	Heterogeneity	
						I^2^ (%)	P_h_
Overall	8	1,904	Random	1.22 (1.04–1.43)	0.02	70	0.001
Region							
Asia	6	1,670	Random	1.25 (1.03–1.52)	0.02	53	0.06
Europe	2	234	Random	1.21 (0.78–1.87)	0.40	80.8	0.02
Treatment							
Surgery	4	823	Random	1.24 (0.95–1.62)	0.11	73.2	0.01
Chemotherapy	2	517	Random	1.26 (0.86–1.84)	0.24	68	0.08
Mixed	2	564	Random	1.25 (0.84–1.85)	0.28	68.9	0.07
Disease stage							
No-metastatic	2	260	Random	1.23 (0.75–2.02)	0.42	77.8	0.03
Mixed (non-metastatic & metastatic)	3	1,003	Random	1.19 (0.89–1.60)	0.24	58.7	0.09
Metastatic	3	641	Random	1.34 (1.01–1.77)	0.04	53.4	0.12
Cut-off for PLR							
>150	2	308	Fixed	1.73 (1.21–2.49)	0.003	36	0.21
≤150	6	1,596	Random	1.13 (0.99–1.30)	0.08	60	0.03
NOS score							
<7	3	444	Random	1.50 (0.85–2.64)	0.16	79	0.008
≥7	5	1460	Fixed	1.18 (1.04–1.33)	0.008	40	0.16

## Discussion

In the present study, we identified 8 studies involving 1,904 patients that investigate the prognostic value of preoperative PLR in patients with PC. This meta-analysis showed that elevated PLR is an independent predictor of worse OS in patients with PC. Furthermore, subgroup analysis showed that the adverse prognostic effect of high PLR remained substantial in Asian studies, patients with metastatic disease and patients with PLR >150, as well as for studies with NOS score ≥7. These findings suggest PLR was predictive of OS. It may provide important supporting information to inform treatment decisions and predict treatment outcomes. For example, clinicians may use different treatment strategies for high-risk patients, such as changes in the inflammatory response, regulation of the immune system, and neoadjuvant chemotherapy, etc. Therefore, the individualized treatment may improve the prognosis of patients with PC.

The actual mechanisms between high PLR and poor outcome of PC are unclear. It has been suggested that cross-talk exists between the inflammatory response and tumor progression [3, 19, 20]. Cancer-related inflammation could suppress antitumor immunity by recruiting immunosuppressive cells such as regulatory T cells and myeloid-derived suppressor cells (MDSC), resulting in tumor progression [21, 22]. Platelet, as a critical source of cytokines, binds directly to members of the VEGF, PDGF, FGF, and TGF-β, thus the platelet acts as a reservoir for secreted growth factors that regulate tumor angiogenesis, cell proliferation, migration, and metastasis [23–25]. Lymphocytes play a critical role in the cell-mediated antitumor immune response. The lymphocyte count reflects the degree of responsiveness of the immune system of the host [26, 27]. Tumor-infiltrating lymphocytes (TILs) are important immune cells found in tumor and responsible for antitumor immune responses [28]. In addition, low lymphocyte counts are thought to be responsible for an insufficient immunological response, which leads to inferior survival in multiple cancers [29, 30]. Taken together, PLR combined with the effects of platelet and lymphocyte may predict the prognosis of patients with PC.

Nevertheless, our study has several limitations. First, excessive heterogeneity existed among studies. Subgroup analyses didn’t find the potential sources of heterogeneity. In addition, we performed sensitivity analysis. The results showed that no study had a significant effect on the observed effect size (pooled HR). Second, the cut-off value for PLR differed in each study. This might be significant contributors to substantial heterogeneity. Third, HRs and their 95% CIs were extracted from univariable analyses in one study and estimated from Kaplan-Meier survival curve in one study. Thus, the prognostic role of PLR might be overestimated. Fourth, all included studies were retrospective, which was more susceptible to some biases. Fifth, although the p values are significant, the 95% CIs for HR for elevated PLR and OS (1.04–1.43), Asian studies (1.03–1.52) and patients with metastatic disease (1.01–1.77) are very close to 1. Given that this is a meta-analysis with a large sample size, and as a result powered to detect small differences, the clinical relevance are of questionable significance.

In conclusion, our findings demonstrated that the preoperative PLR may be a significant independent prognostic factor in patients with PC. However, considering the limitations listed above, future high-quality studies are warranted to further determine the prognostic value of PLR in patients with PC.

## Supporting information

S1 File PRISMA 2009 checklist in this meta-analysis. (DOC)

S2 File Search strategies of this meta-analysis. (DOC)

## References

[1] Siegel R, Naishadham D, Jemal A. Cancer statistics, 2013. CA Cancer J Clin. 2013;63(1):11–30. Epub 2013/01/22. doi: 10.3322/caac.21166 23335087 10.3322/caac.21166

[2] Li D, Xie K, Wolff R, Abbruzzese JL. Pancreatic cancer. Lancet. 2004;363(9414):1049–57. Epub 2004/03/31. doi: 10.1016/S0140-6736(04)15841-8 15051286 10.1016/S0140-6736(04)15841-8

[3] Grivennikov SI, Greten FR, Karin M. Immunity, inflammation, and cancer. Cell. 2010;140(6):883–99. Epub 2010/03/23. PubMed Central PMCID: PMCPmc2866629. doi: 10.1016/j.cell.2010.01.025 20303878 10.1016/j.cell.2010.01.025 PMC2866629

[4] Obata J, Tanaka N, Mizuno R, Kanao K, Mikami S, Matsumoto K, et al Plasma fibrinogen level: an independent prognostic factor for disease-free survival and cancer-specific survival in patients with localised renal cell carcinoma. BJU Int. 2016;118(4):598–603. Epub 2016/01/19. doi: 10.1111/bju.13414 26780664 10.1111/bju.13414

[5] Nakamura K, Nagasaka T, Nishida T, Haruma T, Ogawa C, Kusumoto T, et al Neutrophil to lymphocyte ratio in the pre-treatment phase of final-line chemotherapy predicts the outcome of patients with recurrent ovarian cancer. Oncol Lett. 2016;11(6):3975–81. Epub 2016/06/18. PubMed Central PMCID: PMCPmc4888270. doi: 10.3892/ol.2016.4513 27313726 10.3892/ol.2016.4513 PMC4888270

[6] Facciorusso A, Prete VD, Crucinio N, Serviddio G, Vendemiale G, Muscatiello N. Lymphocyte-to-monocyte ratio predicts survival after radiofrequency ablation for colorectal liver metastases. World Journal of Gastroenterology. 2016;22(16):4211–8. doi: 10.3748/wjg.v22.i16.4211 27122671 10.3748/wjg.v22.i16.4211 PMC4837438

[7] Shirai Y, Shiba H, Sakamoto T, Horiuchi T, Haruki K, Fujiwara Y, et al Preoperative platelet to lymphocyte ratio predicts outcome of patients with pancreatic ductal adenocarcinoma after pancreatic resection. Surgery (United States). 2015;158(2):360–5. 10.1016/j.surg.2015.03.043 26032829

[8] Martin HL. Prognostic value of systemic inflammation-based markers in advanced pancreatic cancer. Intern Med J. 2014. 10.1111/imj.12453 24750233

[9] Asari S, Matsumoto I, Toyama H, Shinzeki M, Goto T, Ishida J, et al Preoperative independent prognostic factors in patients with borderline resectable pancreatic ductal adenocarcinoma following curative resection: The neutrophil-lymphocyte and platelet-lymphocyte ratios. Surg Today. 2016;46(5):583–92. doi: 10.1007/s00595-015-1206-3 26108488 10.1007/s00595-015-1206-3

[10] Sarkut P, Kilicturgay S, Tirnova I. Prognostic role of neutrophillymphocyte and plateletlymphocyte ratio in pancreatic cancer. Hpb. 2016;18:e426–e7.

[11] Dogan M, Algin E, Guven ZT, Baykara M, Kos TF, Uncu D, et al The prognostic significance of neutrophil-lymphocyte ratio, platelet-lymphocyte ratio and prognostic nutritional index in metastatic pancreas cancer. European Journal of Cancer. 2015;51:S429. 10.1080/03007995.2017.1408579 29161926

[12] Kou T, Kanai M, Yamamoto M, Xue P, Mori Y, Kudo Y, et al Prognostic model for survival based on readily available pretreatment factors in patients with advanced pancreatic cancer receiving palliative chemotherapy. Int J Clin Oncol. 2016;21(1):118–25. doi: 10.1007/s10147-015-0864-x 26123314 10.1007/s10147-015-0864-x

[13] Liberati A, Altman DG, Tetzlaff J, Mulrow C, Gotzsche PC, Ioannidis JP, et al The PRISMA statement for reporting systematic reviews and meta-analyses of studies that evaluate health care interventions: explanation and elaboration. PLoS Med. 2009;6(7):e1000100 PubMed Central PMCID: PMCPMC2707010. doi: 10.1371/journal.pmed.1000100 19621070 10.1371/journal.pmed.1000100 PMC2707010

[14] Parmar MK, Torri V, Stewart L. Extracting summary statistics to perform meta-analyses of the published literature for survival endpoints. Stat Med. 1998;17(24):2815–34. 9921604 10.1002/(sici)1097-0258(19981230)17:24<2815::aid-sim110>3.0.co;2-8

[15] Stang A. Critical evaluation of the Newcastle-Ottawa scale for the assessment of the quality of nonrandomized studies in meta-analyses. Eur J Epidemiol. 2010;25(9):603–5. doi: 10.1007/s10654-010-9491-z 20652370 10.1007/s10654-010-9491-z

[16] Tierney JF, Stewart LA, Ghersi D, Burdett S, Sydes MR. Practical methods for incorporating summary time-to-event data into meta-analysis. Trials. 2007;8:16 PubMed Central PMCID: PMCPMC1920534. doi: 10.1186/1745-6215-8-16 17555582 10.1186/1745-6215-8-16 PMC1920534

[17] Egger M, Davey Smith G, Schneider M, Minder C. Bias in meta-analysis detected by a simple, graphical test. BMJ. 1997;315(7109):629–34. 9310563 10.1136/bmj.315.7109.629 PMC2127453

[18] Begg CB, Mazumdar M. Operating characteristics of a rank correlation test for publication bias. Biometrics. 1994;50(4):1088–101. 7786990

[19] Palumbo JS, Degen JL. Mechanisms coupling the hemostatic system to colitis-associated cancer. Thrombosis research. 2010;125 Suppl 2:S39–43. Epub 2010/05/15. 20434003 10.1016/S0049-3848(10)70011-6

[20] Mantovani A, Allavena P, Sica A, Balkwill F. Cancer-related inflammation. Nature. 2008;454(7203):436–44. Epub 2008/07/25. doi: 10.1038/nature07205 18650914 10.1038/nature07205

[21] Brimnes MK, Vangsted AJ, Knudsen LM, Gimsing P, Gang AO, Johnsen HE, et al Increased level of both CD4+FOXP3+ regulatory T cells and CD14+HLA-DR(-)/low myeloid-derived suppressor cells and decreased level of dendritic cells in patients with multiple myeloma. Scand J Immunol. 2010;72(6):540–7. Epub 2010/11/04. doi: 10.1111/j.1365-3083.2010.02463.x 21044128 10.1111/j.1365-3083.2010.02463.x

[22] Wang G, Lu X, Dey P, Deng P, Wu CC, Jiang S, et al Targeting YAP-Dependent MDSC Infiltration Impairs Tumor Progression. Cancer Discov. 2016;6(1):80–95. Epub 2015/12/25. PubMed Central PMCID: PMCPmc4707102. doi: 10.1158/2159-8290.CD-15-0224 26701088 10.1158/2159-8290.CD-15-0224 PMC4707102

[23] Wakefield LM, Smith DM, Flanders KC, Sporn MB. Latent transforming growth factor-beta from human platelets. A high molecular weight complex containing precursor sequences. J Biol Chem. 1988;263(16):7646–54. Epub 1988/06/05. 3163692

[24] Gay LJ, Felding-Habermann B. Contribution of platelets to tumour metastasis. Nat Rev Cancer. 2011;11(2):123–34. Epub 2011/01/25. doi: 10.1038/nrc3004 21258396 10.1038/nrc3004 PMC6894505

[25] Banks RE, Forbes MA, Kinsey SE, Stanley A, Ingham E, Walters C, et al Release of the angiogenic cytokine vascular endothelial growth factor (VEGF) from platelets: significance for VEGF measurements and cancer biology. British journal of cancer. 1998;77(6):956–64. Epub 1998/04/07. PubMed Central PMCID: PMCPmc2150108. 9528841 10.1038/bjc.1998.158 PMC2150108

[26] Kitayama J, Yasuda K, Kawai K, Sunami E, Nagawa H. Circulating lymphocyte is an important determinant of the effectiveness of preoperative radiotherapy in advanced rectal cancer. BMC Cancer. 2011;11:64 Epub 2011/02/11. PubMed Central PMCID: PMCPmc3041780. doi: 10.1186/1471-2407-11-64 21306650 10.1186/1471-2407-11-64 PMC3041780

[27] Ceze N, Thibault G, Goujon G, Viguier J, Watier H, Dorval E, et al Pre-treatment lymphopenia as a prognostic biomarker in colorectal cancer patients receiving chemotherapy. Cancer chemotherapy and pharmacology. 2011;68(5):1305–13. Epub 2011/03/31. doi: 10.1007/s00280-011-1610-3 21448592 10.1007/s00280-011-1610-3

[28] Man YG, Stojadinovic A, Mason J, Avital I, Bilchik A, Bruecher B, et al Tumor-infiltrating immune cells promoting tumor invasion and metastasis: existing theories. J Cancer. 2013;4(1):84–95. Epub 2013/02/07. PubMed Central PMCID: PMCPmc3564249. doi: 10.7150/jca.5482 23386907 10.7150/jca.5482 PMC3564249

[29] Hoffmann TK, Dworacki G, Tsukihiro T, Meidenbauer N, Gooding W, Johnson JT, et al Spontaneous apoptosis of circulating T lymphocytes in patients with head and neck cancer and its clinical importance. Clinical cancer research: an official journal of the American Association for Cancer Research. 2002;8(8):2553–62. Epub 2002/08/13. 12171883

[30] Vayrynen JP, Tuomisto A, Klintrup K, Makela J, Karttunen TJ, Makinen MJ. Detailed analysis of inflammatory cell infiltration in colorectal cancer. British journal of cancer. 2013;109(7):1839–47. Epub 2013/09/07. PubMed Central PMCID: PMCPmc3790164. doi: 10.1038/bjc.2013.508 24008661 10.1038/bjc.2013.508 PMC3790164

