# Supplementary material for: Preoperative platelet lymphocyte ratio as independent predictors of prognosis in pancreatic cancer: A systematic review and meta-analysis
Source: PLoS One. 2017 Jun 2;12(6):e0178762. doi: 10.1371/journal.pone.0178762 (PMC5456351; doi:10.1371/journal.pone.0178762)
Supplement: S2 File — (DOC) [file pone.0178762.s002.doc]

**Chochrane:**

#1 (PLR or platelet to lymphocyte ratio or platelet lymphocyte ratio or platelet-lymphocyte ratio)

#2 (pancreas)

#3 (pancrea*)

#4 MeSH descriptor: [Pancreas] explode all trees

#5 #2 or #3 or #4

#6 MeSH descriptor: [Carcinoma] this term only

#7 MeSH descriptor: [Adenocarcinoma] this term only

#8 MeSH descriptor: [Carcinoma, Ductal] this term only

#9 MeSH descriptor: [Neoplasms] explode all trees

#10 (cancer* or carcin* or neoplas* or tumo* or cyst* or growth* or adenocarcin* or malig*)

#11 #6 or #7 or #8 or #9 or #10

#12 #5 and #11

#13 (survival or prognosis or recurrence or clinical outcome)

#14 #1 and #12 and #13

**MEDLIME (Ovid SP):**

1 (PLR or platelet to lymphocyte ratio or platelet lymphocyte ratio or platelet-lymphocyte ratio).mp

2. (pancreas or pancrea*).mp.

3. exp Pancreas/

4. 3 or 2

5. Carcinoma/

6. Adenocarcinoma/

7. Carcinoma, Ductal/

8. exp Neoplasms/

9. (cancer* or carcin* or neoplas* or tumo* or cyst* or growth* or adenocarcin* or malig*).mp.

10. 5 or 6 or 7 or 8 or 9

11. 4 and 10

12 (survival or prognosis or recurrence or clinical outcome).mp

13 1 and 11 and 12
